# Progress on a Carbon Nanotube Field-Effect Transistor Integrated Circuit: State of the Art, Challenges, and Evolution

**DOI:** 10.3390/mi15070817

**Published:** 2024-06-25

**Authors:** Zhifeng Chen, Jiming Chen, Wenli Liao, Yuan Zhao, Jianhua Jiang, Chengying Chen

**Affiliations:** 1 School of Opto-Electronic and Communication Engineering, Xiamen University of Technology, Xiamen 361024, China; hahahamm1232023@163.com (Z.C.);; 2 School of Electronics, Peking University, Beijing 100871, China

**Keywords:** CNTFET, compact model, integrated circuits, ballistic transport

## Abstract

As the traditional silicon-based CMOS technology advances into the nanoscale stage, approaching its physical limits, the Carbon Nanotube Field-effect Transistor (CNTFET) is considered to be the most significant transistor technology beyond Moore’s era. The CNTFET has a quasi-one-dimensional structure so that the carrier can realize ballistic transport and has very high mobility. At the same time, a single CNTFET can integrate hundreds of nanowires as the conductive channels, enabling significant current transport capabilities even in low supply voltage, thereby providing a foundational basis for achieving nanoscale ultra-large-scale analog/logic circuits. This paper summarizes the development status of the CNTFET compact model and digital/analog/RF integrated circuits. The challenges faced by SPICE modeling and circuit design are analyzed. Meanwhile, solutions to these challenges and development trends of carbon-based transistors are discussed. Finally, the future application prospects of carbon-based integrated circuits are presented.

## 1. Introduction

As the traditional silicon-based transistor devices scale down to below 3 nm, the three-dimensional transistor structures such as FinFET and Gate All Around (GAA) encounter challenges in effectively controlling the conductive channel due to leakage current, edge parasitic capacitor, source/drain resistor, tunneling current, and other effects. These limitations significantly constrain the advancement of process nodes [1]. To sustain integrated circuit technology beyond Moore’s era, the Carbon Nanotube Field-effect Transistor (CNTFET) emerges. The CNTFET exhibits superior bandgap and carrier transport characteristics [2]. The manufacturing process of CNTFET is compatible with existing mature silicon-based processes, eliminating the need for redevelopment of complex production and manufacturing procedures. This greatly reduces the difficulty of device development and the research cycle. Therefore, CNTFET has become a potent contender for the next generation of nanoscale transistors.

Based on whether the terminal contact of the channel belongs to a metal–semiconductor property, CNTFETs are classified into Schottky barrier CNTFET and quasi-MOS CNTFET. The electronic advantages of CNTFET consist of high mobility, large current-carrying capacity, and superior channel electrostatic control. Within CNTFETs, electron conduction involves a complex mechanism due to the random network distribution of carbon nanotubes. Unlike single-layer graphene transistors (GFETs) where electron motion is strictly confined to a single atomic plane, CNTFETs exhibit both intra-tube and inter-tube conduction pathways. Different carbon nanotubes may not necessarily align along a single direction, and superpositions of nanotubes can occur, allowing conduction across different planes of carbon. This quasi-one-dimensional structure endows CNTFETs with unique electrical characteristics [3]. In an ideal scenario, charge carriers undergo minimal wide-angle scattering, with only forward and backward scattering resulting from electron–phonon interactions. This signifies that charge carriers exhibit ballistic or quasi-ballistic transport within the channel, characterized by an exceedingly long mean free path (MFP); CNTFETs exhibit extremely high room-temperature carrier mobility and saturation velocity [4]. Experimental data indicate that at room temperature, the carrier mobility in CNTs can reach up to 1 × 10^5^ cm^2^/(V·s), which is approximately 100 times that of silicon, and the saturation velocity can reach 4 × 10^7^ cm/s, about 4 times that of silicon, without velocity overshoot. For field-effect transistors, carrier mobility and saturation velocity are critical parameters. In a single CNTFET, it is possible to integrate hundreds of nanowires as conductive channels, significantly boosting its current-carrying capability. The operational principle of the CNTFET resembles that of conventional silicon devices. This device is switched on or off through electrostatic control of the gate. The quasi-one-dimensional structure of the CNTFET allows for superior gate electrostatic control in the channel region compared to that of three-dimensional and two-dimensional devices (such as FD-SOI).

In conventional Tunneling Field-effect Transistors (TFETs), current must tunnel through the barrier, causing significant resistance and limiting current amplitude. A carbon heterojunction from partially unzipped metallic CNTs (m-CNTs) significantly reduces the tunneling barrier, achieving a larger on-state current. Both ends of an m-CNT are partially unzipped into semiconducting armchair graphene nanoribbons (AGNRs), which are used as the source and channel, thereby diminishing the tunneling barrier [5]. Yu Zhu utilized a unique all-carbon AGNR-ZGNR-CNT heterojunction to design a barrier-free TFET. Through self-consistent first-principle DFT-NEGF calculations, it was demonstrated that this design can reduce the subthreshold swing below the classical limit and increase the on-state current by diminishing the tunneling barrier [6]. Qing You developed high-performance photodetectors by forming a SWCNTs/graphene all-carbon heterojunction [7]. They achieved a photoresponsivity increase from 1091 A/W to 2842 A/W at 590 nm and from 314 A/W to 1043 A/W at 940 nm, along with a response time reduction from 40.94 ms to 7.39 ms, compared to traditional SWCNTs-based photodetectors. This enhancement provides a pathway for high-performance, miniaturized, large-scale, and broadband photodetectors. Daeyeon Koh introduced a fully inkjet-printed flexible photodetector (PD) array [8]. This photodetector array leverages a unique heterostructure that combines carbon nanotubes (CNTs) with photoactive organic macrocycles (OMs), endowing the PD array with color selectivity. The integration of CNTs and OMs facilitates the transfer of photo-excited carriers into the CNT channel, thereby generating a photocurrent.

The rapid development of CNTFET has led to the emergence of research interests in CNTFET-based digital/analog/RF integrated circuits, and System on Chip (SoC). However, most of the achievements are limited to simulations or small-scale integrated circuits. Among them, compact models and standard Process Design Kits (PDK) remain pressing issues in CNTFET research. Compact models are essential prerequisites for large-scale simulation and verification of CNTFET integrated circuits. Compact models are generally categorized into three types: numerical models, empirical models, and macro models. In order to meet the requirements of ultra-large/extreme-scale integrated circuit simulations, CNTFET compact models need to consider ultra-short channel effects, scattering/reflection mechanisms, tunneling mechanisms, interface trap states, and more [9]. Furthermore, mathematical models within compact models must maintain strict continuity to attain analytical solutions for electrical parameters in various operating regions, thereby ensuring better convergence in simulations. In contrast to mature silicon-based SPICE models, numerical computations within carbon-based compact models are notably more complex. The extensive use of multiplication and integration operations can lead to a sharp increase in simulation time and reduced convergence. As a result, a trade-off between model accuracy and simulation complexity must be struck by constructing numerical/semi-empirical compact models [10,11]. In recent years, the academic community has presented some accomplishments in the realm of carbon-based logic circuits, SRAM, and radio frequency integrated circuits. However, these achievements have yet to meet the production standards for standard large-scale industrialization. The absence of a standard PDK primarily constrains the simulation of large-scale integrated circuits [12]. The currently published carbon-based circuits are mostly simulated based on behavioral models before fabrication, with limitations on device performance uniformity and scalability.

In CNTFET research, ensuring the reliability of compact models’ predictive results is crucial. To validate the accuracy and applicability of these models, researchers typically employ comprehensive evaluation methods, including comparison with experimental data, device level, and integrated circuit level validations. These results are tested under extreme operating conditions, and compared with other existing models. Comparative analysis with experimental data involves verifying the consistency between model predictions and actual experimental results, while device-level validation assesses the model’s performance in specific circuit configurations, such as inverters and amplifiers. At the integrated circuit level, the model is used to simulate entire CNTFET integrated circuits. These simulations demonstrate the potential of new technologies and support the design process, helping to optimize performance before fabrication. So, digital, analog, and RF circuits are adopted to evaluate their applicability in large-scale integration. Testing under extreme operating conditions focuses on the unique properties of CNTFETs, such as short-channel effects and tunneling mechanisms. Lastly, comparing the model with other existing models aids in assessing its relative advantages and limitations. The comprehensive use of these validation methods helps thoroughly evaluate and ensure the effectiveness of CNTFET compact models in the design and simulation of large-scale integrated circuits, thereby advancing the industrial application of carbon-based semiconductor devices. This paper will provide an overview of CNTFET compact models and circuits, analyze existing research achievements and identified issues, and ultimately discuss the future trends and applications of carbon-based integrated circuits.

## 2. Current Research Status

### 2.1. CNTFET Compact Model

As in Table 1, currently, compact models for CNTFETs mainly include analytical models and semi-empirical Virtual-source (VS) models. Analytical models utilize the non-equilibrium Green’s function method (NEGF) to obtain precise analytical solutions, enabling accurate descriptions of device mechanisms and performance. However, the analytical equations are often intricate and computationally demanding, occasionally leading to difficulties in obtaining exact analytical solutions, resulting in non-convergence issues during circuit simulation. The VS model combines analytical equations, test data, and fitting parameters. It assumes that most of the channel carriers occupy lower energy levels. The channel length is comparable to or smaller than the carrier scattering MFP so that non-elastic scattering can be neglected. It also employs segmental approximation for different operational regions. As a result, a semi-empirical compact model compatible with SPICE-like simulators has been established.

Most of the early proposed compact models for CNTFETs were based on non-equilibrium Green’s function method [13,14,15,16]. These compact models are based on device testing results and assume that CNTFETs possess ideal ballistic transport channels while neglecting carrier tunneling effects. Moreover, these compact models only account for single-gate conductive channel CNTFET structures and fail to capture current crosstalk and screening effects between CNTFETs. Liu et al. developed a transport model for CNTFETs based on the non-equilibrium Green’s function method. This discards the crude classical continuum model and directly links the transport characteristics of CNTFETs to the chiral indices of CNTs. Soheli Farhana proposed a transport phenomenon for CNTFETs using the NEGF method, connecting CNT chiral vectors directly with CNTFET transport properties and realizing the ballistic CNTFET through carrier transport in the conduction and valence bands. From the simulated results, the on-state current behavior of semiconducting CNTs was observed to be 69.5 μA [17,18]. Therefore, these simplifications lead to inaccuracies when evaluating transistor transient responses and dynamic performance. When the characteristic dimensions of CNTFETs are smaller than the anticipated MFP in the model, the carrier mobility decreases, and the dynamic performance is far from what the simulated structure suggests. On the other hand, due to the extensive use of integration functions for modeling calculations, such compact models are relatively inefficient in simulations and are prone to encountering non-convergent states.

**Figure 1:**
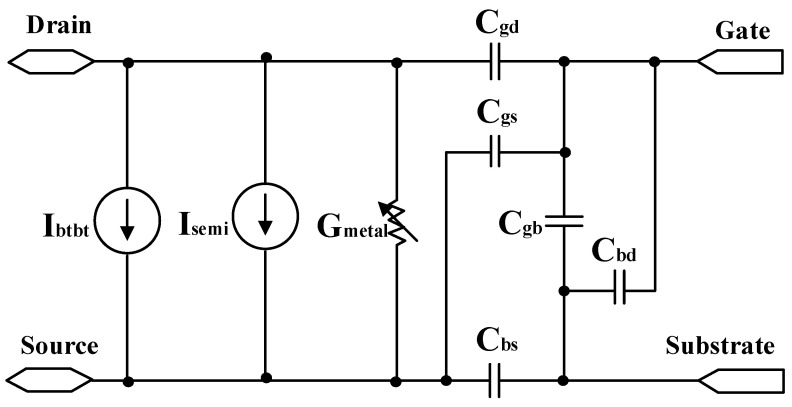
Compact CNTFET model proposed by the Stanford University research team.

Ref. [19] introduced the first comprehensive 32 nm CNTFET compact model [13]. As shown in Figure 1, the model encompasses key small-signal and large-signal parameters: drain current (Isemi), source–drain resistor (Gmetal), tunneling current Ibtbt, and capacitors between various terminals (Cgd/Cgs/Cgb/Cbd/Cbs). The model is implemented using the Verilog-A hardware language and supports the HSPICE simulation software. It includes axial/radial quantum confinement effects, acoustic/optical phonon scattering effects, and screening effects between multiple nanowires. Targeting digital standard cell applications, this achievement also introduces a gate delay evaluation metric, CV/I, for comparison with silicon-based transistor performance.

Building upon the research findings from ref. [13], the compact model introduced in Ref. [20] incorporates channel elastic scattering effects, drain/source series resistor, Schottky barrier resistor, and parasitic gate capacitor, significantly enhancing the model’s physical characteristics. However, this achievement employs a simplified energy band structure, restricting the model’s application in scenarios requiring high power supply and high surface potentials (>>1.0 eV). Additionally, the diffusion capacitance arising from minority carriers at the source/drain junctions is disregarded, impacting the accuracy of AC response simulation in RF circuits and constraining the model’s application in RF integrated circuits [21,22]. Wei et al. extensively investigated the mechanism of quantum capacitor generation in carbon nanotube (CNT) and its influence on dynamic performance, refining the transport characteristics of charge carriers within the conductive channel. However, this model assumes that internal electrical parameters are independent of CNT diameter, leading to significant discrepancies between simulation results and experimental data [23].

Targeting the 9 nm process node application, J. Luo et al. proposed a semi-analytical compact model for CNTs based on the VS model [24]. The model consists of a series resistor, parasitic capacitor, and direct source–drain tunneling leakage. The model was calibrated using corresponding experimental data, and the results indicated that the contact resistor is a critical limiting factor in performance. Due to the low effective mass in CNTs, direct source–drain tunneling can lead to severe leakage, thereby inhibiting further reduction of the gate length. The main contribution of this achievement lies in exploring the design space to minimize gate delay under constraints of leakage current in the off state. It attempts to balance parasitic effects by optimizing the lengths of the gate, contact region, and extension region.

**Figure 2:**
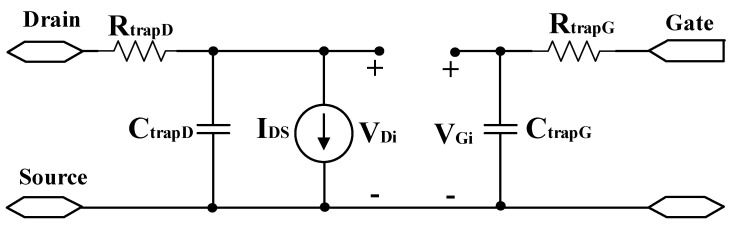
Depicts a compact model incorporating trap effects.

Compact models proposed in refs. [25,26], based on 15 nm device testing results, intricately describe the intrinsic current–voltage and charge–voltage characteristics of CNTFETs. This model includes the relationship between carrier effective mobility and diameter. It deduces short-channel effects, such as subthreshold slope degradation and drain-induced barrier lowering. The model also investigates the impact of parasitic effects leading to size scaling and performance degradation. It is employed to study the trade-off between driving current and leakage current based on the selection of diameter, density, contact length, and target contact–gate spacing. Other research endeavors have investigated Schottky barriers and surface defects within CNTs, and have introduced delay resistors (RtrapG/RtrapD) and capacitors (CtrapG/CtrapD) to characterize trap effects [27,28,29], as shown in Figure 2.

Zhang et al. initially proposed a method that combines the subband equation with diameter distribution to calculate the current for individual subbands [30]. The model also employs a segmented approximation to divide the subband energy into three segments, with the average energy within each segment used to calculate the respective contributing current. Eventually, the total current contribution ratio for each segment is computed using a probability density function. This compact model avoids the need for integration calculations during simulations, thereby enhancing simulation efficiency. When extending the model, the accuracy of the current can be improved by increasing the number of segments. However, a limitation lies in the neglect of proximity and coupling effects among multiple CNTs.

Based on bulk silicon MOSFET test data, ref. [31] introduces a simplified X-VS compact model. This model comprises only a few key model parameters, enhancing simulation flexibility and speed. It not only characterizes CNTFETs but can also be applied to InGaAs HFETs. However, its limited parameters can only represent the fundamental intrinsic characteristics of CNTFETs and fail to account for factors like tunneling current and parasitic effects, leading to significant discrepancies with actual device performance.

Due to the significant influence of thermal effects on the high-frequency (HF) electrical characterization of CNTFETs, ref. [32] combines the high-frequency admittance parameters-based method and trap-free characterization technique to establish a lumped-element electro-thermal model for multitube CNTFETs. Unlike the traditional four-terminal gate resistor technique, this model employs a two-port network to extract the short thermal time constant. For the first time, this model confirms the thermal stability of multitube CNTFETs.

### 2.2. CNTFET Digital Integrated Circuit

The performance metrics of digital integrated circuits are presented in Table 2. Digital signals are less susceptible to noise, interference, and attenuation, making digital circuits more reliable for data transmission and storage. Additionally, digital circuits exhibit strong error tolerance to noise and transmission errors. CNTs, as an emerging nanomaterial, are bringing fresh opportunities and immense potential to the development of digital integrated circuits. Guided by carbon nanotube technology, their extremely small size (diameter of 1–3 nm), ultra-high intrinsic mobility for both electrons and holes (greater than 1 × 10^5^ cm^2^/(V·s)), and long carrier mean free path (typically greater than 1 μm) make them an ideal choice for digital integrated circuit design. In recent years, carbon nanotube field-effect transistors have been widely utilized in the realm of digital circuits, including fundamental logic gates like inverters and NAND gates, as well as processors and other digital circuitry.

In 2014, Ronak S. Oswal et al. introduced a digital event-counting comparator circuit [33]. The study compared the power consumption and delay of designs based on CNTFETs and 32 nm CMOS technology, leveraging the effective use of multi-threshold logic design in CNTFETs, where the threshold voltage is dependent on the diameter of the CNT used, as opposed to MOSFETs, which require various doping layers to modify the device’s threshold voltage. The employed CNTFET model was derived from the Verilog-A representation of the Stanford compact model. Comprehensive simulation results revealed significant reductions in power consumption and shorter circuit delay time when using CNTFETs for circuit implementation. Multi-threshold logic design can be effectively implemented in CNTFETs since the threshold voltage depends on its diameter, whereas in MOSFETs, various doping layers are required to modify the device’s threshold voltage. The threshold voltage of a CNTFET is governed by its diameter, which is determined by its chirality.

Leveraging the property of threshold voltage modulation through CNTFET diameter control, in 2019, JABER et al. utilized the diameter-dependent threshold voltage characteristics of CNTFET to optimize standard three-valued logic gates, including inverter, three-valued NAND gate, and three-valued half-adder circuits. This approach reduced the number of transistors used, employed an energy-efficient transistor layout, and facilitated dual-supply voltage operation [34]. Furthermore, Abdelrahman’s research revealed that the threshold voltage of CNTFETs is determined by the diameter of CNTs, which is suitable for multi-threshold digital circuit design. Building upon this principle, they designed a ternary multiplier and compared it with traditional silicon-based binary circuits in terms of power consumption, area, and delay time [35]. The comparison is performed in terms of area, power, delay, and power delay product versus variation of supply voltage and temperature. The study primarily focuses on the performance of carbon nanotube field-effect transistors (CNTFETs) under varying operating temperatures. Virtual Source CNTFETs (VS-CNTFETs) with the Stanford model are simulated using HSPICE, investigating supply voltages of 0.8 V, 0.9 V, and 1 V, as well as temperatures of −40 °C, 27 °C, and 85 °C. The results indicate that with increasing supply voltage and temperature, there is a corresponding increase in power consumption and a decrease in delay. Through an analysis of the power-delay product (PDP), it is observed that the specific circuit exhibits superior performance across different supply voltages and temperatures. This suggests that carbon nanotubes demonstrate a degree of robustness under varying temperature conditions.

In 2020, Amr Mohammaden et al. utilized memristors and CNTFETs to implement various ternary full adders [36]. Additionally, memristors can be stacked over CNTFETs to reduce chip area. They proposed the realization of a 4-bit Carry Ripple Adder, Carry Skip Adder, and Carry Lookahead Adder. A comparative study was conducted on them under different supply voltages and temperatures. The Carry Lookahead Adder exhibited the best performance in terms of power-delay product (PDP), making it the optimal design among the three types of full adders. By using the VTEAM memristor model, they provided a detailed description of the device’s actual performance, including the capability to switch threshold voltage. In the same year, Mostafa Parvizi et al. introduced a high-speed, low-power majority logic-based CMFA (Current Mode Full Adder) circuit [37]. The proposed CMFA consists of only 14 transistors and employs 32 nm CNTFETs based on the Stanford model. Simulations were executed at a voltage of 0.5 V, operating frequency of 1 GHz, load capacitance of 2 fF, and current of 10 µA. This CMFA is suitable for reference current sources in low-voltage, high-speed applications.

### 2.3. CNTFET Analog Integrated Circuits

The performance metrics of analog integrated circuits are presented in Table 3. In certain applications involving continuous signal processing and precise control, analog circuits still possess unique advantages compared to digital circuits. As an emerging nanomaterial, CNTs bring forth new opportunities and potential for the development of analog circuits. Leveraging CNT technology, with its exceptional electrical performance, high energy efficiency, and size advantages, has positioned it as an ideal choice for analog circuit design. In recent years, CNTFETs have found prominent applications in fundamental analog circuits such as operational amplifiers, second-order filters, comparators, and oscillators.

In 2015, Puri et al. designed a low-power operational amplifier (Op-Amp) and sample-and-hold circuit with CNTFETs [38]. Simulations were performed using HSPICE software, comparing various performance parameters of Op-Amp circuits based on CNTFETs and MOSFETs. The analysis indicated that the CNTFET-based circuit exhibited lower power consumption and superior performance. By utilizing CNTFETs, the proposed Op-Amp demonstrated a remarkable 80.8% reduction in power consumption, while the sample-and-hold circuit’s power consumption was reduced by 71.14%. In 2018, Puttananjegowd et al. proposed and implemented a cascode common-source multistage transconductance amplifier (TIA) circuit using CNTFET technology [39]. Experimental results demonstrate that by considering an appropriate number of CNTs, spacing, and diameter, the performance of the proposed TIA can be enhanced, achieving ultra-low-power, low-noise transimpedance amplification. Utilizing CNTFET technology enables the circuit to attain improved conductivity, low noise, and low power consumption advantages, thereby significantly boosting transimpedance gain, bandwidth, and overall performance. In the same year, Choudhary et al. utilized SPICE simulations to compare the performance of analog-to-digital converters (ADCs) based on MOSFET and CNTFET [40]. The results indicate that at low supply voltages, with similar linearity, the CNTFET-based ADC exhibits lower power consumption and reduces non-linearity errors, particularly demonstrating better performance at lower resolutions. This study provides a foundation for future higher-resolution ADC designs.

In 2019, Jogad et al. proposed a method for constructing filters using CNTFET and Current-mode Second-generation Current Conveyors (CCII) [41]. This approach is innovative in enhancing bandwidth, reducing power consumption, and minimizing harmonic distortion. Through detailed simulations and performance analyses, the superiority of the CNTFET-CCII filter was demonstrated in terms of current and voltage bandwidth, impedance, power consumption, and distortion, compared to traditional CMOS-CCII designs. In 2020, Pankaj Singla et al. proposed a novel approach for low-power level shifters [42]. The circuit was simulated and verified at the 32 nm technology node, and its performance was compared to circuits using other technologies such as MOSFET and FINFET. Simulation results indicated that voltage level shifters implemented using CNTFET technology exhibited lower static power consumption and leakage power compared to other technologies. Shailendra et al. demonstrated the AC gain of single-stage, two-stage, and three-stage common-source amplifiers through detailed simulation analysis, showcasing variations under different parameter settings [43]. The research findings highlighted the significant influence of parameters such as the number of CNT channels, diameter, oxide thickness, and dielectric constant on the operational amplifier gain.

### 2.4. CNTFET Memory

In recent years, the challenges faced by modern memory technologies have become increasingly prominent. With the scaling of feature size, issues related to leakage power and stability have become focal points of research. In this context, CNTFETs have emerged as promising alternatives to CMOS technology. Against this backdrop, Mohita et al. conducted a comparative analysis between CNTFET technology and traditional CMOS technology for the 6T SRAM cell [44]. This in-depth analysis delved into critical performance aspects such as leakage power, delay, and stability across different technology nodes. The study confirmed the potential of CNTFETs in optimizing SRAM performance, providing new insights for future memory designs. Elangovan et al. focused on the stability analysis of the 6T SRAM cell based on CNTFETs [45]. A comparison was made between the stability of the 6T CNTFET SRAM cell using a single-nanotube CNTFET and multiple-nanotube CNTFETs. The study also considered the impact of different chirality on stability. The research findings indicated that the 6T CNTFET SRAM based on a single nanotube and low chirality vector exhibited excellent stability across various operating modes.

Shrivastava et al. proposed the use of CNTFETs as an alternative technology to address the challenges of designing SRAM at submicron scales [46]. The paper extensively compares the performance of SRAM under different process conditions, various chirality, and voltage conditions with multiple types of SRAM cells. In the same year, Chauhan et al. designed efficient and low-power SRAM cells using CNT technology to meet the growing demands of data centers [47]. Researchers leveraged the advantages of CNT technology to innovatively reduce energy consumption and enhance speed. By comparing the performance of various SRAM cells, they highlighted the energy efficiency and speed advantages of SRAM based on CNT technology.

In this research area, Ashish Sachdeva et al. proposed a novel 8-bit SRAM cell design, known as the BLP8T cell [48]. This design innovatively introduced the concept of bitline-powered operation. By optimizing circuit design and utilizing CNTFETs, it achieved improved performance in low-power applications, including reduced leakage power, enhanced access time, and improved stability parameters. This study not only offers an efficient SRAM cell option for low-power devices but also further underscores the potential and prospects of CNTFET technology in the field of memory.

## 3. Challenges Faced by CNTFET Integrated Circuits

After nearly 20 years of development, CNTFET has demonstrated significant growth potential and commercial value. However, the compact model, digital, analog, and memory circuits of this device are still in the exploratory stage, with some fundamental issues related to device operation mechanisms, non-ideal factors, and performance parameters that need to be addressed. The authors believe that the practical realization of the modeled and simulated devices must be included. Furthermore, the practical realization of CNTFET-based circuits is fraught with challenges that stem from the fabrication process. These include issues like achieving uniformity in CNT synthesis and integration with existing semiconductor technologies. Addressing these fabrication-related concerns is essential for transitioning from theoretical models and simulations to high-performing, real-world integrated circuits. Without solving these practical issues, the commercial viability of CNTFETs remains uncertain.

### 3.1. Challenges in CNTFET Compact Modeling

Currently, the known compact models for CNTFETs assume one-dimensional ballistic/quasi-ballistic transport in the conducting channel, where carriers undergo no scattering or reflection during transport. However, in practice, when the channel length exceeds MFP, thermally excited carriers at the transport terminal may experience optical and acoustic phonon scattering. Additionally, when carrier energy falls below the drain potential barrier, reflection phenomena can occur. Furthermore, traps generated by surface defects can randomly capture and release carriers. These non-ideal effects can lead to an actual drain current significantly lower than the theoretically predicted value. Therefore, it is necessary to comprehensively consider these factors and establish corrective terms in compact models for drain current, accounting for the various limiting factors, to match theoretical and practical values. Furthermore, severe tunneling effects exist in CNTFETs. The band-to-band tunneling current constitutes a significant proportion of the overall current in the device. Therefore, to assess the subthreshold behavior and static power consumption of the device, it is essential to incorporate the formulation of the band-to-band tunneling current into the compact model of the device [19,20]. Similar to bulk silicon MOSFETs, CNTFETs also include a multitude of parasitic resistors and capacitors [24]. The series resistor is composed of the drain/source extension resistor (Rext) and the contact resistor (Rc) between the CNT and the metal contact [48,49,50]. Rext is directly proportional to the ratio of the channel length to MFP, while the contact resistor is related to the injected carriers at the metal contact, scattering probability, and device doping conditions. The parasitic capacitor includes the fringe capacitor (COF) between the gate and the outer edge of the source/drain extension in CNTFET and the coupling capacitor (CGTP) between the gate and the source/drain contact body [51]. Parasitic resistors and capacitors not only slow down the switching speed but also increase dynamic power consumption. Therefore, an accurate assessment of parasitic resistance and capacitance is essential in the compact model to objectively reflect key performance metrics of the actual device, such as speed and power consumption.

### 3.2. Challenges in CNTFET Digital Integrated Circuits 

Within the domain of digital integrated circuits based on CNTFETs, the current focal point primarily resides in circuits comprising smaller-scale modules, such as the realization of logic gates like inverters, adders, and multipliers. However, for the purpose of substantiating the performance of CNTFETs, it becomes imperative to transition from individualized modules to the substantiation of multi-level logic system circuits [52]. Exploring more complex system-level circuits and application scenarios to validate the performance of CNTFET in different contexts requires in-depth research into the advantages and characteristics of CNTFET technology, in order to better leverage its electrical properties.

The main purpose of Multiple-valued Logic (MVL) is to enhance the information transmission capability of digital signals within a system. By employing circuits based on MVL, it is possible to reduce power consumption and interconnect count under certain circumstances [53]. Currently, in the field of digital integrated circuits based on CNTFETs, ternary logic is primarily employed for circuit design due to its potential to reduce power consumption and enhance integration density. However, as demands for integrated circuit performance continue to increase, seeking innovative validation methods becomes crucial. To verify the performance of CNTFETs and enable broader applications, the introduction of quaternary logic or other logic modes becomes a plausible approach. These novel logic modes can further optimize circuit power consumption and delay, fostering the development and innovation of the CNTFET digital integrated circuit domain.

Apart from the challenges in the field of multi-valued logic circuits, there is also relatively limited work comparing CNTFETs with other types of nanowire transistors. The significance of such comparisons lies in the fact that different nanowire transistor technologies possess distinct electrical characteristics, performances, and application potentials. By contrasting CNTFETs with these diverse nanowire transistor technologies, we can comprehensively assess the performance of CNTFETs across various technological aspects.

### 3.3. Challenges in CNTFET Analog Integrated Circuits

CNTFET devices possess unique electrical characteristics, such as hysteresis induced by various traps, which can impact their performance. To achieve acceptable performance in amplifiers, it is necessary to address issues like device instability and leakage current. Further optimization of the high-frequency performance of CNTFETs is required to meet broader application requirements [54]. For applications demanding high-gain devices, CNTFETs have emerged as an ideal choice. Their low input-referred noise levels (typically around 0.5–1 µV/√Hz), ultra-low power consumption (in the range of a few nanowatts), and exceptionally high common-mode rejection ratios (exceeding 100 dB) support these applications [55,56]. Additionally, CNTFETs offer fast response times (in the order of picoseconds) and wide bandwidths (up to several hundred GHz), which make them suitable for high-frequency applications [57]. The primary drawbacks of employing CNTFETs lie in their lower unit-gain bandwidth and poorer transient performance, rendering them unsuitable for high-frequency applications [58]. Due to the limitation of its unit gain bandwidth, the response of CNTFET devices in the high-frequency range is constrained, making the design of high-performance RF analog circuits more challenging. In high-frequency applications, circuits require a fast transient response and wide bandwidth, and the characteristics of CNTFETs might restrict their performance in this regard.

CNTFETs hold promising prospects in the field of analog circuits, particularly due to their significant advantages in low power consumption and high integration density. However, due to inherent differences in physical properties and manufacturing processes compared to traditional electronic components, there are relatively few demonstrated CNTFET analog circuit cases. Similar to CNTFET digital integrated circuits, analog circuit design in CNTFET analog integrated circuits is often limited to simple circuits such as operational amplifiers and filters, while more complex analog circuits are not fully supported by current processes. There are limitations of CNTFETs in terms of low-power and high-resolution sensor interfaces. Traditional CMOS processes perform poorly under energy efficiency and high-resolution requirements, especially in low supply voltage and high noise environments. Consideration must be given to noise and other issues in the design.

### 3.4. Challenges in CNTFET Memory

In the fields of memory integrated circuits and digital integrated circuits, CNTFETs face challenges associated with scaling feature sizes, increasing leakage currents, and decreasing reliability. Issues related to internal and inter-chip interconnects in memory chips designed based on traditional binary logic have become prominent in binary circuitry. Future research needs to focus on the applications of CNTFET circuits in multi-valued logic and non-volatile memory to meet the growing demands for computation and storage.

With the advancement of chip processing capabilities, not only the design of memory is affected, but also the layout and wiring issues of chip components are becoming more prominent. In this rapidly evolving context, the performance and characteristics of CNTFETs are also evolving. Therefore, models need to be constantly updated to adapt to the new device characteristics. This involves the complexity of CNTFET models and the intricacies of parameter extraction processes. The application of Stanford’s CNTFET model in digital circuit design might prove overly intricate, making it challenging for widespread adoption in practical applications.

Currently, CNTFET technology is predominantly focused on the implementation of SRAM. As technology progresses, there is a need to extend CNTFET technology to other memory architectures. While the application of CNTFET in SRAM has brought numerous advantages such as lower power consumption and improved performance, different memory possess distinct challenges and requirements in terms of architecture and operation. By applying CNTFET technology to other memory architectures, not only can memory performance be enhanced, but also the overall advancement of memory technology can be propelled. Therefore, the expansion of CNTFET technology from SRAM to other memory architectures is a promising avenue of research.

## 4. Corresponding Solutions

### 4.1. Compact Model Solutions

In CNTFETs, it can be assumed that carriers undergo ballistic or quasi-ballistic transport, and the drain–source current can be expressed as follows [59,60]:

(1) $$\begin{array}{l} I=\frac{4ek_{B}T}{h}\sum_{p=1}^{+\infty }[\ln(1+\exp\frac{{eV}_{CNT,Si}sbbd[p]}{k_{B}T})\\ -\ln(1+\exp\frac{{eV}_{CNT,Di}-sbbd[p]}{k_{B}T})] \end{array}$$

In Equation (1), the first term represents the effect originating from source to source Fermi level, while the second term represents the effect from drain to drain Fermi level. Similarly, due to the quantum mechanical nature of charge in CNTFETs and neglecting carrier scattering, the drain current can be calculated using the non-equilibrium Green’s function. This is a good approximation for CNTFETs operating at low bias voltages [61,62].

Saurabh Sinha et al., considering the conditions of drain and source potential barriers, incorporated the effect of source–drain junction reflection. They introduced the transmission coefficient T(E) for carriers passing through the two potential barriers [63]. Due to the attenuation rate of the thin oxide layer, the potential barrier takes on an approximate triangular shape. Consequently, the exponential potential distribution is approximated as a triangular distribution, leading to the derivation of the analytical expression for tunneling probability [64,65]. By solving the tunneling probability equation at the source and drain contacts, the final expression for drain current considering tunneling probability is obtained:

(2) $$\begin{array}{l} I=\frac{4q}{h}\sum_{n}\int_{En}\mathrm{sgn}(E)T(E)[F(\mathrm{sgn}(E)(E,\mu_{s}))\\ +F(\mathrm{sgn}(E),(E,\mu_{s}-V_{ds}))]dE \end{array}$$

In Equation (2), the value of as 1 represents the conduction band, and the value of −1 represents the valence band, denotes the Fermi–Dirac integrals, and represents the Fermi level at the source.

Fregonese et al. conducted a detailed analysis of the Schottky barrier model in CNTFETs and segmented the channel into source–channel, intrinsic inner-channel, and drain–channel sections [66]. The charge in each region is obtained by integrating the product of the Density of States (DOS) and the corresponding Fermi distribution over the energy range, ultimately leading to the drain current based on the obtained charge values.

Lundstrom et al. proposed a method for addressing partial ballistic transport characteristics in conventional MOSFETs. This method includes the effects of backscattering [67]. This approach can serve as a correction to the ballistic model, and its advantage lies in offering a straightforward and comprehensive strategy. Building upon this theoretical foundation, Paolo Michetti et al. proposed a semi-analytical model for CNTFETs [68]. In this model, the non-ballistic transport channel is regarded as a combination of N ballistic channel segments, where N is the ratio of the channel length to MPF. In the transport state, a transistor with Schottky barrier contacts is modeled as a series of individual ballistic channels connected in series. The head and tail of this concatenated ballistic channel are linked to the source and drain through Schottky barrier contact, with the boundaries fixed at μ_0_ = μ_s_ and μ_N_ = μ_d_, where μ_s_ and μ_d_ represent the Fermi levels of the source and drain, respectively. Within the Nth ballistic channel, μ_n−1_ and μ_n_ serve as the source and drain Fermi levels for the sub-channel, and once the channel potential is determined, the current for the Nth channel can be obtained. It is important to note the distinction between the internal channel within the ballistic chain and the boundary channels with source and drain. In fact, the characteristics of the first and last ballistic channel are Schottky barrier contacts with metal source and drain, while the internal channel can be seen as an ohmic transistor. In the ohmic-contacted ballistic chain composed of N channels, after linearization, the current can be arranged in a form similar to drift-diffusion, and its current can be expressed as follows:

(3) $$I_{ds}=\frac{q^{2}\Gamma (1)\ell }{\pi \hbar L}\sum_{\alpha }\int_{V_{s}}^{V_{d}}\left\{F_{-1}(\eta_{\alpha }^{e}[V])-F_{-1}(\eta_{\alpha }^{h}[V]\right)\}dV$$

where $F_{-1}(x)$ is the Fermi–Dirac integral of order −1, and Γ is the gamma function. Additionally:

(4) $$\eta_{\alpha }^{e}=(q\phi_{c}-qV-\varepsilon_{\alpha })/kT$$

(5) $$\eta_{\alpha }^{h}=(-q\phi_{c}-qV-\varepsilon_{\alpha })/kT$$

where $\phi_{c}$ represents the electrostatic potential of the CNTFET, and $\varepsilon_{\alpha }$ represents the edge energy level of the *α*-th subband. In the case of an extremely long channel, relative to MPF, the drain current is limited by the central drift-diffusion transistor. This model characterizes the transition from barrier-limited to channel-limited transport mechanisms for carrier transport. This semi-analytical model presents a precise and straightforward approach to capturing the physical characteristics of CNTFETs with Schottky barrier contact at very low computational cost.

Igor Bejenari et al. considered the effects of acoustic and optical phonon scattering in channels, assuming an exponential decay of the channel potential [69]. They subsequently used the Fermi–Dirac distribution function and a simple approximation for the transmission probability to derive an analytical expression for the Landauer–Buttiker drain current. This model has largely overcome the limitations of assuming ideal ballistic transport in previous compact models, resulting in an effective analytical model formula that significantly enhances convergence during simulations.

In the early stages, most compact models of CNTFETs utilized the Wentzel–Kramers–Brillouin (WKB) approximation to obtain the band-to-band tunneling probability, denoted as $T_{\mathrm{btbt}}$, and incorporated this probability into the analytical expression of the drain current Id [70]. Guojun Zhu et al. proposed a Miller–Good (MG) tunneling model [71]. This model exhibits a smoother transition near the hot electron and tunneling transition region compared to the WKB model. However, the MG model does not actually address the inherent inaccuracies of the WKB tunneling model.

### 4.2. Solution for CNTFET Digital Integrated Circuits

When facing the challenges of CNTFET digital integrated circuits, innovative solutions need to be sought to drive performance verification in multi-level logic systems and further developments in broader application domains. While the current focus is primarily on the implementation of small modular circuits such as inverters, adders, and multipliers, in order to comprehensively validate the performance of CNTFETs, we must broaden our perspective to encompass multi-level logic circuits and explore more complex applications.

**Figure 3:**
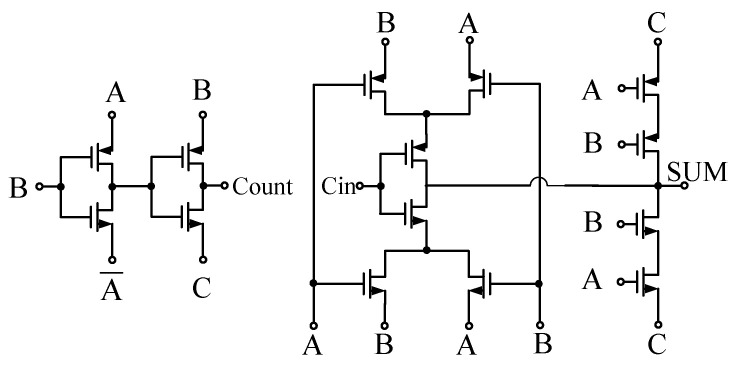
Pass transistor-based full adder.

Amarnath et al. designed a low-power RISC-V processor using a Pass-transistor Logic (PTL) architecture based on CNTFETs [72], which is illustrated in Figure 3. Despite the traditional preference for complementary logic over Pass-transistor Logic (PTL) in silicon-based designs, CNTFETs, due to their low threshold voltage, and low power consumption are ideal candidates for PTL. By incorporating PTL design modules in critical path components of the processor, the advantages of CNTFETs in terms of stability and low power consumption in large-scale circuits have been demonstrated. Gadgil et al. proposed a novel 2-bit ternary arithmetic logic unit (TALU) design based on CNTFETs [73]. This design employed a simplified 2:1 multiplexer approach, eliminating the need for input decoders and thus streamlining the circuit. By introducing an output multiplexer, outputs from different operations could be merged into one, offering potential for applications in processors. This design could be further extended to higher-bit TALU designs, providing a robust option for future multi-valued logic circuit applications.

**Figure 4:**
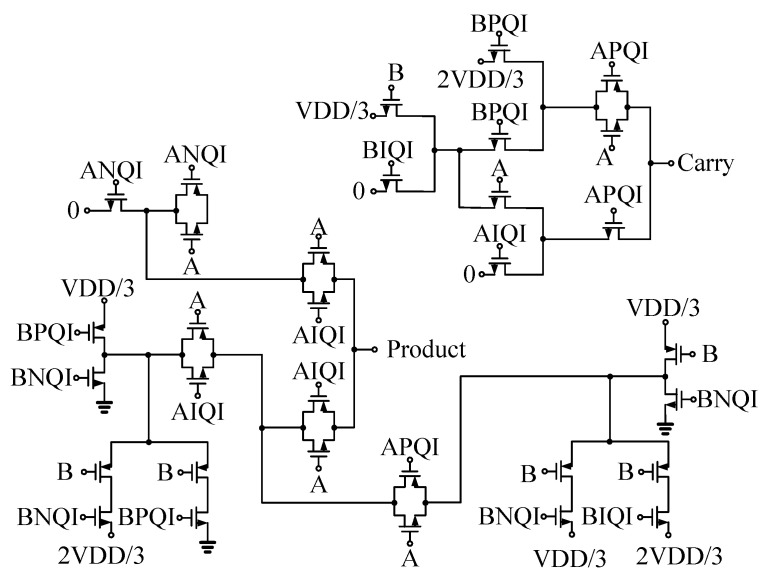
Quaternary One Digit Multiplier.

Patel et al. proposed a novel design optimization method for quaternary logic circuits, introducing innovative approaches for quaternary half-adders, full-adders, and quaternary multipliers. Figure 4 represents a Quaternary One Digit Multiplier. In the first part, B, BNQI, BPQI, and BIQI serve as inputs to the transistor gates, and logic values are conveyed using individual transistors. In the second part, A, ANQI, APQI, and AIQI are employed as inputs to the transistor gates, while the incoming logic values from the first part are routed through a 2:1 MUX. In their method, a pair of transistors in the logic gate were replaced by a single N-type or P-type CNTFET transistor as the switching element for circuit propagation logic [74]. They adjusted the chirality of the transistor to fine-tune the threshold voltage, optimizing circuit performance while reducing the required transistor count and overall power consumption. On the other hand, Yadav et al. introduced a dual-mode logic design based on CNTFET for AND gates, OR gates, and multiplexers. They introduced additional transistors and logic paths, enabling the circuit to operate in both static and dynamic modes to achieve a better tradeoff of energy efficiency and performance [75].

Furthermore, during the exploration of solutions, it is crucial to compare CNTFET with various nanoscale transistors. Different nanoscale transistor technologies possess unique electrical characteristics, performance, and potential applications. Venkataiah C designed a low-power, high-speed 4-input adder and compared it with a ternary adder using Graphene Nanoribbon FETs (GNRFETs) [76]. Simulation results demonstrated that circuits designed with CNTFET exhibited favorable performance in terms of power consumption, carry propagation delay, and power-delay product. On the other hand, Erfan Abbasian compared GNRFET and CNTFET devices based on their characteristics related to threshold voltage, GNR width, and CNT diameter [77]. They proposed an efficient ternary multiplier (TMUL), and the results indicated that CNTFET had advantages over GNRFET in terms of delay improvement and power reduction. Both studies revealed that CNTFET not only outperforms CMOS devices but also demonstrates similar performance superiority when compared to other nanoscale transistors.

### 4.3. Solution for CNTFET Analog Integrated Circuits

To address the limitations of CNFET in high-frequency applications, it is essential to further enhance its high-frequency performance. This can be achieved by exploring methods such as improving device structures or optimizing circuits to enhance the response speed and bandwidth of CNFET in the high-frequency range. Currently, the design of complex analog circuits based on CNTFET is constrained by manufacturing processes. However, innovative design approaches can be explored to implement some complex analog circuits at the current process level. Through careful circuit topology and optimized design, better performance can be achieved with limited resources.

Masud et al. incorporated CNTFETs into a novel all-pass filter (APF) topology for low-voltage, low-power, and high-frequency fully differential applications. They introduced a fully differential first-order all-pass filter circuit based on CNTFETs. Tunability of the pole frequency within the range of 10.5 to 26 GHz was achieved by adjusting the variable capacitor based on CNTFET [78]. This circuit not only exhibits superior performance in terms of pole and power consumption but also proves suitable for low-voltage environments, showcasing excellent robustness. Silva et al. proposed two versatile high-frequency circuit designs: a Phase Configurable Amplifier (PCA) and a Frequency Configurable Amplifier (FCA) [79]. By fully leveraging the bipolar characteristics of CNTFETs, they successfully achieved various circuit operation modes under different bias conditions, including in-phase amplifiers, out-of-phase amplifiers, and frequency multipliers. Employing consistent matching and stability networks, particularly in the case of the FCA, they accomplished frequency doubling, achieving a significant harmonic suppression effect of up to 20 dB. This achievement provided crucial support for the stable high-frequency operation of the circuit. Building upon these versatile circuit designs, further modulation schemes such as Phase Shift Keying (PSK) and Frequency Shift Keying (FSK) were realized.

With the advancement of technology, the challenge of cross-process integration needs to be addressed to enable the coexistence of CNTFET circuits with traditional CMOS circuits. Gielen, based on Stanford University’s compact model technique, designed a capacitive sensor interface for a Bang-Bang Phase-Locked Loop (BBPLL) with a 1 μm process [80]. This design is constructed by an oscillator and a digital control section. The circuit achieves the digitalization of infrared signals by modulating the oscillation frequency. This design demonstrates the feasibility of using CNTFETs for time-based sensor interfaces, providing insights for future integration of CNTFETs with sensors. Rebecca Ho et al. first proposed a complete complementary analog circuit based on CNFETs [81]. The circuit starts with fundamental operational amplifier building blocks, gradually extends to CNFET-based sensor interface circuits, and finally achieves a physical demonstration. This work successfully integrated CMOS circuits with CNFET gas sensors. Tamersit et al. demonstrated the detection of DNA molecules by introducing DNA molecules into nanogaps and utilizing the electrical characteristics of CNTFETs [82]. This provides a direction for the future development of nanobiosensing technology.

### 4.4. Solution for CNTFET Memory

Multi-valued logic can reduce interconnect complexity and area overhead, as circuits designed in multi-valued logic can enhance the performance of various arithmetic and digital signal processing applications. Additionally, multi-valued circuits offer simplicity and higher energy efficiency in memory designs. Karthikeya integrated ternary logic with CNTFETs [83], utilizing CNTFETs as fundamental components to leverage their attributes, such as low power consumption, high speed, and compact size. They designed a ternary SRAM, demonstrating the synergy between ternary logic and CNTFETs. This research achievement encompasses circuit design and simulation, development of control and storage units, and performance evaluation. The outcomes of this study offer a design approach that combines ternary logic with CNTFET-based SRAM, providing a novel direction for future low-power and high-efficiency processor designs. Sonal Shreya proposed a novel approach by integrating CNT ternary SRAM with phase change random access memory (PCRAM) [84], aimed at addressing the challenge of high current density requirements in PCRAM. This integrated design holds the potential to optimize PCRAM performance and mitigate the risk of current leakage.

In order to facilitate the widespread use of CNTFETs in circuit applications, a corresponding compact model is urgently needed. Spasova proposed a simplified model based on the original Stanford model, effectively reducing the number of model parameters [85]. This simplified model was verified through simulations in digital memory circuitry, demonstrating its practical applicability. Furthermore, the study also showcased a 16-bit DRAM memory circuit with a 1T DRAM cell and a 2-bit decoder. The circuit was implemented using both the compact model from Stanford University and the derived simplified CNTFET model. By comparing the simulation results of the two models, the reliability and suitability of the simplified model in memory circuitry were further confirmed.

Zahoor utilized a Verilog-A model based on RRAM devices in their study [86], which featured bipolar switching characteristics. To simplify the analysis, the design ignored variations in the Stanford model for RRAM devices. By replacing traditional MOSFETs, CNTFETs were introduced as selector devices into the ITIR RRAM domain. Analysis of individual memory cells and memory arrays demonstrated the advantages of the CNTFET-RRAM design over traditional designs, including lower power consumption and higher performance. This research paves the way for future memory applications, highlighting the potential of CNTFETs in the realm of RRAM.

## 5. Future Application Prospects

As researchers delve deeper into the exploration of CNTFET integrated circuits in the fields of digital, analog, and memory technologies, the research potential and commercial value of CNTFET integrated circuits are gradually becoming apparent. Considering the research directions, CNTFET integrated circuits are expected to find applications in the following areas:

Imaging Systems: In reference [7], constructing SWCNTs/graphene heterojunctions significantly enhances the photoresponsivity of photodetectors to 2842 A/W (at 590 nm) and 1043 A/W (at 940 nm). This all-carbon heterojunction facilitates the efficient separation and transport of photogenerated carriers, paving the way for high-performance, miniaturized, large-scale, and broadband photodetectors. Similarly, reference [8] highlights a unique heterostructure combining carbon nanotubes (CNTs) with photoactive organic macrocycles (OMs) to enable color selectivity. A proof-of-concept color detection system demonstrated distinct signal patterns for each tested wavelength, effectively distinguishing light colors. This demonstrates the potential for mass-producible photodetector arrays in applications such as wearable optoelectronic sensing and imaging systems.

Medical Electronics: In ref. [79], the proposed DNA nanosensor is endowed with a sensitive channel based on a zigzag carbon nanotube (Z-CNT). The obtained results empower the proposed nanobiosensor to be considered a promising candidate for high-performance label-free DNA sensing applications at the nanoscale domain, where the dimensions of bio-measurands are comparable with those of nanobiosensors. The small size and excellent conductivity of CNTs enable the design of more compact and efficient medical sensors. These sensors can achieve highly sensitive monitoring of biological signals, physiological parameters, and disease biomarkers. This will provide patients with more accurate medical data, aiding in early diagnosis, treatment, and monitoring.

Sensor Technology: CNT sensors can be used to monitor changes in gas concentrations in the air, chemical contents in liquids, and variations in environmental temperature. Their high sensitivity makes these sensors promising for applications in environmental monitoring, industrial control, and medical diagnostics. In ref. [78], the integration of CNTFET technology in sensor designs, such as the aforementioned TIA for medical diagnostics, highlights their potential to create more sensitive and efficient sensing devices.

High-performance Computing and Processor Technology: In reference [36], it has been articulated that CNTFETs offer the potential for low power consumption and size reduction. Building on these properties, CNTFETs show immense potential in the realms of high-performance computing and processor technology. The electronic properties of CNT materials allow CNTFET integrated circuits to remain stable at high frequencies while generating less heat. This positions them as promising candidates for high-performance computing and chip technologies. By leveraging CNTs, it is possible to develop faster, more efficient, and lower-energy-consuming devices.

## 6. Conclusions

CNTFETs represent a significant advancement in transistor technology, especially as traditional silicon-based CMOS technology approaches its physical limits. The unique quasi-one-dimensional structure of CNTFETs enables the ballistic transport of carriers, resulting in high mobility and substantial current transport capabilities even at low supply voltages. This makes CNTFETs a foundational technology for developing nanoscale ultra-large-scale analog and logic circuits, essential beyond Moore’s era. This paper has reviewed the current research status of CNTFETs, including their compact models, digital and analog integrated circuits, and memory applications, each presenting distinct challenges. However, promising solutions are emerging, demonstrating the robustness and adaptability of CNTFET technology. We have also discussed the future application prospects of carbon-based integrated circuits, emphasizing their potential to revolutionize electronics. Despite existing challenges, the continuous advancement of CNTFET technology and innovative solutions pave the way for their integration into mainstream electronic devices, offering enhanced performance and efficiency.

## Figures and Tables

**Table 1:** The pros and cons of compact models of CNTFET.

Model Type	Advantages	Disadvantages
Non-equilibrium Green’s Function Method	Accurate analytical solutions, in-depth analysis of CNTFET electrical behavior.	Computational complexity, not suitable for large-scale circuit simulations.
Semi-empirical Compact Models	Suitable for fast circuit simulations, high computational efficiency. Reduces complexity through simplified assumptions.	Coarse performance description, lacks in-depth understanding. Relies on empirical assumptions, may not accurately capture device details.

**Table 2:** Performance metrics of digital integrated circuits.

Performance Metric	Description
Power Consumption	Low power is a critical performance metric in digital circuits.
Speed	High switching speed is crucial for digital circuits.
Integration Level	Integrating more circuits in a small space is an advantage.
Logic Gate Delay	Key performance metric, especially in computation and control.
Multi-value Logic Support	CNTFET may support multi-value logic designs, such as ternary or quaternary.

**Table 3:** Performance metrics of analog integrated circuits.

Performance Metric	Description
Power Consumption	Analog circuits require low power, typically quantified by power dissipation in milliwatts (mW).
High-frequency Performance	Crucial for analog circuits in communication and RF applications, measured by transit frequency (f_t) and maximum oscillation frequency (f_{max}).
Interference Resistance	Analog circuits need to perform well in noisy environments, quantified by signal-to-noise ratio (SNR) in decibels (dB).
Linear Performance	Good linear performance is essential, especially in signal processing and amplification, measured by compression gain and total harmonic distortion (THD).
Cross-process Integration	Carbon-based analog circuits need to collaborate with traditional CMOS circuits, measured by integration efficiency and compatibility metrics.
Sensor Interface Performance	For specific applications, analog circuits may need to provide specific sensor interface performance.

## Data Availability

All the data are reported/cited in the paper.
